# Vasodilator Effects of Quercetin 3-O-Malonylglucoside Are Mediated by the Activation of Endothelial Nitric Oxide Synthase and the Opening of Large-Conductance Calcium-Activated K^+^ Channels in the Resistance Vessels of Hypertensive Rats

**DOI:** 10.3390/molecules30132867

**Published:** 2025-07-06

**Authors:** Maria Luiza Fidelis da Silva, Erdi Can Aytar, Arquimedes Gasparotto Junior

**Affiliations:** 1 Laboratory of Cardiovascular Pharmacology (LaFaC), Faculty of Health Sciences, Federal University of Grande Dourados (UFGD), Dourados 79.804-070, MS, Brazil; m.alufidelis@hotmail.com; 2 Department of Horticulture, Faculty of Agriculture, Usak University, 64200 Uşak, Türkiye; erdicanaytar@gmail.com

**Keywords:** flavone, mesenteric vascular bed, K^+^ channels, vasodilation

## Abstract

We used molecular docking as a computational tool to predict the binding affinities and interactions of quercetin 3-O-malonylglucoside (Q3MG) with vascular target proteins. First, the proteins 1M9M (human endothelial nitric oxide synthase; eNOS) and 6ND0 (human large-conductance voltage- and calcium-activated K^+^ channels; BK_Ca_) were downloaded from the Protein Data Bank and submitted to molecular docking studies, revealing Q3MG binding affinities for both proteins. The vascular effect of Q3MG was investigated in the perfused mesenteric vascular beds (MVBs) of spontaneously hypertensive rats. In preparations with functional endothelium, Q3MG dose-dependently reduced the perfusion pressure in MVBs. Removal of the endothelium or inhibition of the nitric oxide synthase enzyme by L-NAME blocked the vasodilation induced by Q3MG. Perfusion with a physiological solution containing high KCl or the use of a non-selective blocker of K^+^ channels, as well as perfusion with iberiotoxin, completely abolished the vasodilatory effects of Q3MG. The data obtained suggest that the vascular effects of Q3MG involve the activation of the NO/cGMP pathway followed by the opening of BK_Ca_.

## 1. Introduction

Cardiovascular diseases (CVDs) are the leading cause of deaths worldwide, accounting for approximately 30% of all global deaths [1]. Among CVDs, hypertension stands out as it affects one in every three adults worldwide. It is a chronic condition in which blood pressure remains persistently elevated in the arteries. The main risk factors for hypertension include heredity, obesity, physical inactivity, smoking, excessive alcohol consumption, stress, high intake of sodium chloride, and saturated fats. Uncontrolled hypertension can lead to serious complications, including heart attacks, heart failure, strokes, and kidney failure [2].

Controlling hypertension involves weight loss, regular physical exercise, reducing sodium and fat intake, increasing the consumption of fruits, vegetables, and legumes, quitting smoking, and reducing alcohol consumption. There are several pharmacological therapeutic options for controlling hypertension, notably beta-blockers, calcium channel blockers, diuretics, ACE inhibitors, angiotensin II receptor AT1 antagonists, and aldosterone synthase inhibitors [3].

Despite well-defined treatment, the control of hypertension is inadequate worldwide at around 10%. The main cause is low adherence to pharmacological and non-pharmacological treatments, influenced by habits, behaviors, values, and beliefs [4]. In this context, various countries promote traditional medicine as a complementary treatment for hypertension, with medicinal plants and their metabolites being the primary form of intervention [5]. Although there is extensive scientific literature supporting the use of natural products in hypertension treatment, there remain numerous unexplored compounds, leaving an important gap regarding their effectiveness and safety [6].

Among the classes of secondary metabolites of plants with medicinal interest, flavonoids stand out. Flavonoids are a large group of polyphenolic compounds widely distributed in vegetables, herbs, and fruits [7]. Studies indicate that flavonoids can exert different effects on the cardiovascular system, including vasodilatory, antihypertensive, antioxidant, antiatherosclerotic, and cardioprotective activities [8].

**Figure 1:**
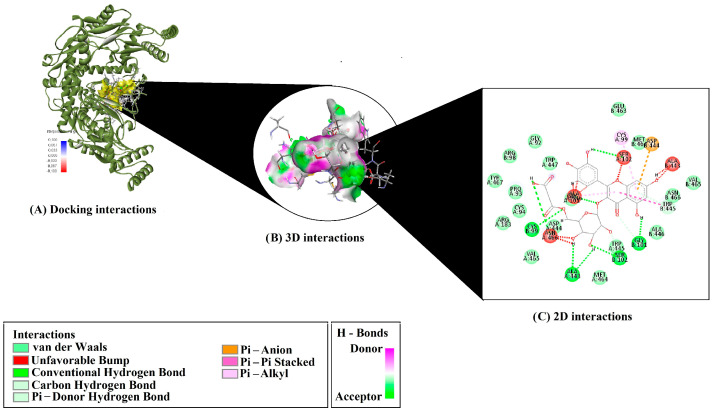
3D and 2D interaction diagrams of Q3MG with the 1M9M protein (CYP51). (**A**) Docking pose of Q3MG in the binding pocket of CYP51 (PDB: 1M9M). (**B**) 3D interaction surface showing van der Waals forces, hydrogen bonds, and π interactions. (**C**) 2D interaction map with key residues; green and pink lines indicate H-bonds, while red, orange, and violet lines show unfavorable π–anion and π–π stacking interactions.

One of the most studied plant metabolites worldwide is quercetin, a major representative of the flavonol group of flavonoids. Although the studied form is the aglycone (the non-sugar part of the glycoside), quercetin is found in plant foods in a glycosylated form and accumulates as glucuronide and sulfate conjugates in the blood circulation [9]. The flavonoid quercetin 3-O-malonylglucoside (Q3MG; Figure 1A), a conjugate of quercetin, is one of the less studied glycosylated derivatives. Various plant species produce and store Q3MG, including green-leafed lettuce (*Lactuca sativa*) [10], *Sicana odorífera* [11], and *Morus alba* [12]. Pharmacological studies on the flavonoid Q3MG are quite limited and involve plant species that synthesize and store this flavonoid in their parenchyma. Thus, despite reports of antioxidant [11], antiatherogenic [12], and hypoglycemic [13] activities, the effects of Q3MG on peripheral vascular resistance remain unknown. In this study, we used molecular docking as a computational tool to predict the binding affinities and interactions of Q3MG with vascular target proteins. Furthermore, we investigated the vasodilatory properties of Q3MG on the peripheral vascular resistance of hypertensive rats, exploring the pharmacological mechanisms involved.

## 2. Results

### 2.1. Molecular Docking

The molecular docking results demonstrated that Q3MG exhibited strong binding affinities toward both 1M9M and 6ND0 target proteins, with binding energies of −9.3 kcal/mol and −9.4 kcal/mol, respectively. The corresponding ligand efficiency (LE) values were calculated as 0.238 for 1M9M and 0.241 for 6ND0, indicating efficient binding in relation to molecular size. Fit quality (FQ) scores of 0.829 (1M9M) and 0.838 (6ND0) further supported the favorable interaction profiles. The binding efficiency index (BEI) values were determined to be 0.016 for 1M9M and 0.017 for 6ND0, while the estimated inhibition constants (Ki) were 0.151 μM and 0.128 μM, respectively.

Detailed interaction profiling of Q3MG revealed extensive non-covalent interactions within the binding sites of both 1M9M and 6ND0 proteins (Table 1). For the 1M9M complex, Q3MG formed multiple conventional hydrogen bonds with key residues including ALA446, CYS99, SER102, GLY101, and ASN466. Additional C–H bonding interactions were observed with GLY101, SER102, and ASN466. A notable π–donor hydrogen bond was established with TRP445, which also contributed to π–π stacking interactions (two occurrences). Pi–alkyl interactions with ALA446 and CYS99 further stabilized the binding configuration (Figure 1).

**Figure 2:**
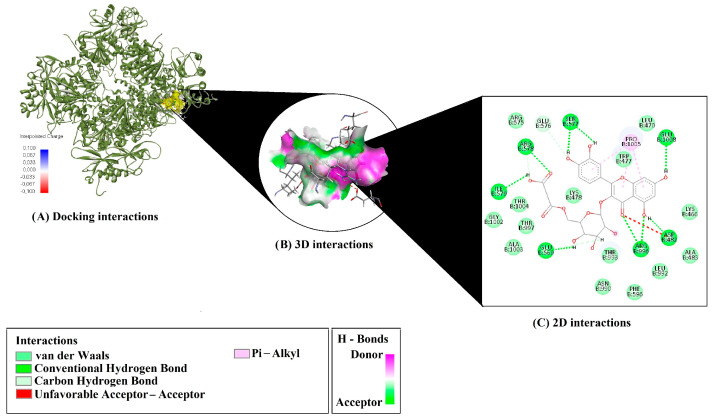
3D and 2D interaction diagrams of Q3MG with the 6ND0 protein. (**A**) Docking pose of Q3MG in the active site of 6ND0. (**B**) 3D surface view highlighting hydrogen bonds and π–alkyl contacts. (**C**) 2D interaction diagram with involved residues; red lines indicate unfavorable interactions; green and pink lines show H-bond acceptors/donors.

In the 6ND0 complex, Q3MG engaged in numerous conventional hydrogen bonds with residues such as ARG578, ARG996, GLU569, ASP482, GLU1008, SER577, and ILE579. C–H interactions were also detected, involving GLU576, ARG578, and GLU569. A single intramolecular π–sigma interaction was observed within the ligand structure, while π–alkyl interactions were formed with PRO1005 (three occurrences), suggesting a stable hydrophobic contribution to the overall binding (Figure 2). These findings highlight the diverse and robust binding mechanisms of Q3MG with both protein targets, supporting its potential as a versatile bioactive molecule.

### 2.2. Vasodilator Effects of Q3MG on the MVBs of Hypertensive Rats

**Figure 3:**
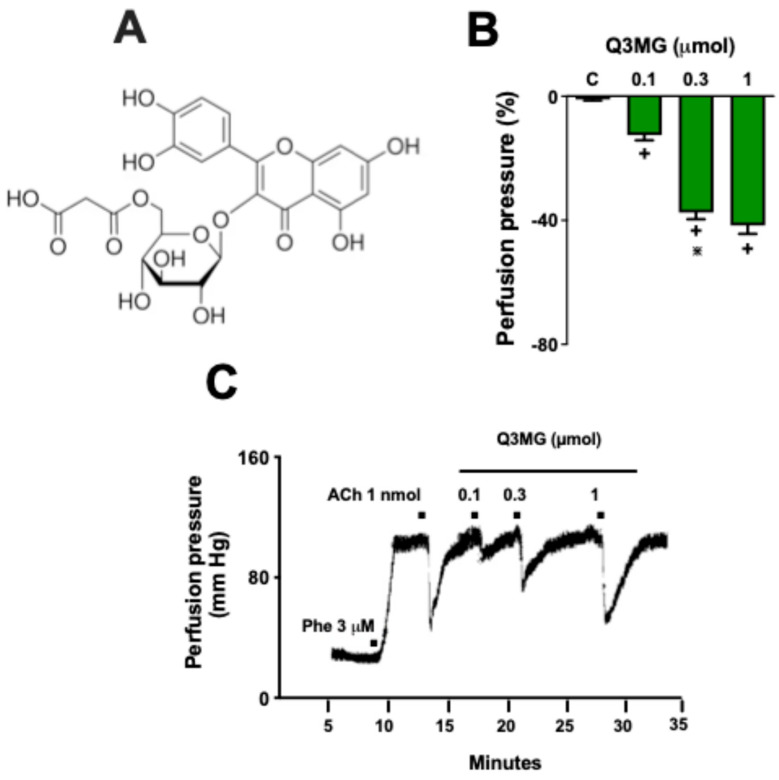
Q3MG promotes dose-dependent vasodilation in the mesenteric vascular bed of rats (MVBs). The chemical structure of Q3MG is shown in panel (**A**). The effects of Q3MG on the perfusion pressure in the MVBs are presented in panel (**B**). Trace recording of mesenteric vascular perfusion pressure showing the effects of acetylcholine (ACh) and Q3MG administration (**C**). Values in panel (**B**) are expressed as mean ± S.E.M. of six different preparations. The comparison with the control group and between the different doses of Q3MG was conducted through one-way ANOVA followed by the Bonferroni test. **^+^** indicates *p* < 0.05 compared with the control group (vehicle administration [PSS] in pre-contracted MVBs with Phe 3 µM). **^⋇^** Indicates *p* < 0.05 compared with Q3MG 0.1 µmol. All experiments were performed in endothelium-intact preparations.

The continuous perfusion of Phe (3 µM) was able to induce a sustained increase in the perfusion pressure in the MVBs, ranging from 27 ± 3.9 mm Hg to approximately 112 ± 12.5 mm Hg. Under these conditions, the administration of 1 nmol of ACh reduced the perfusion pressure by 45 ± 6 mm Hg, demonstrating the viability of the preparations. The administration of Q3MG (Figure 3A) induced a significant and dose-dependent vasodilation, confirmed by the reduction in perfusion pressure recorded in the isolated organ bath apparatus (Figure 3B). It is worth noting that the vasodilation induced by a single dose of the highest dosage of Q3MG (1 μmol) induced a similar response to that obtained after an injection of 1 nmol of acetylcholine (ACh; ~40 mm Hg) (Figure 3C).

### 2.3. The Vascular Effect of Q3MG Is Dependent of Vascular Endothelium and NO/cGMP Pathway

**Figure 4:**
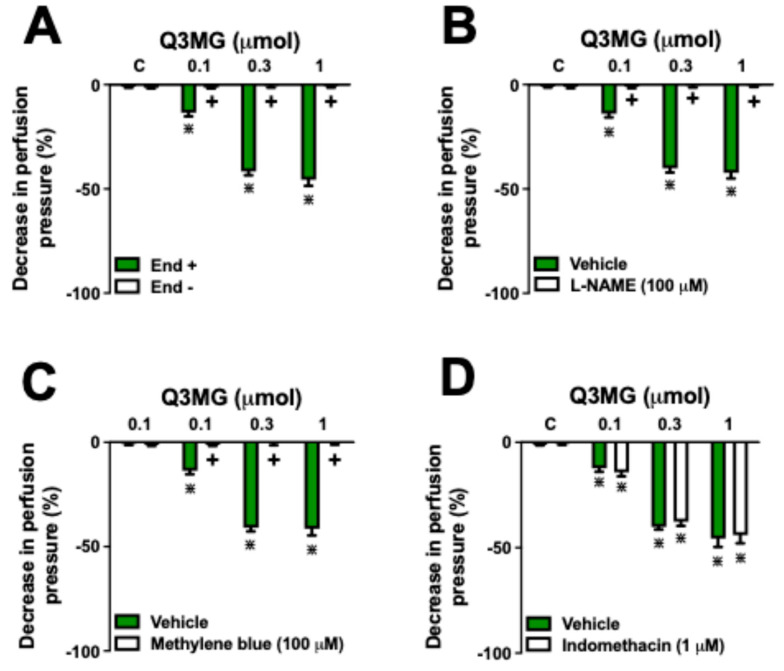
Role of endothelium and the nitric oxide/cGMP pathway on the vasorelaxant effect of Q3MG. Effects of Q3MG on endothelium-intact (End+) and endothelium-denuded (End-) preparations (**A**), or End+ MVBs continuously perfused with L-NAME (**B**), methylene blue (**C**), or indomethacin (**D**). The results show the mean ± S.E.M. of 6 preparations per group. The comparison between the different doses of Q3MG was conducted through one-way ANOVA followed by the Bonferroni test. The difference between the administration of inhibitors/antagonists and the perfusion with the vehicle only, at each dose, was performed using the Student’s *t*-test. **^+^** Indicates *p* < 0.05 compared with the effects of Q3MG on the End+ (**A**) or the vehicle group (**B**–**D**). **^⋇^** Indicates *p* < 0.05 compared with the respective control group. The vehicle represents the perfusion with only the physiological saline solution. All experiments were performed in the mesenteric vascular bed pre-contracted with PSS containing Phe 3 µM.

Treatment with sodium deoxycholate reduced the effects of ACh 1 nmol on the mesenteric vascular perfusion pressure by 95 ± 5%, confirming the efficacy of the chemical removal of endothelial function. Similarly, the chemical removal of the endothelium also reduced the vasodilatory effects of Q3MG (0.1, 0.3, and 1 µmol) by 97 ± 3% (Figure 4A). Moreover, the effects of all Q3MG doses were reduced by 98 ± 2% after prior perfusion with L-NAME (a non-selective nitric oxide synthase inhibitor; Figure 4B) and by 99 ± 2% with methylene blue (MB; an inhibitor of guanylate cyclase; Figure 4C). Preincubation with indomethacin (a non-selective inhibitor of cyclooxygenases) did not result in any significant effect on the vasorelaxant response to Q3MG, excluding the participation of prostaglandins in the vascular effects (Figure 4D).

### 2.4. The Vascular Effect of Q3MG Involves Activating BK_Ca_ Channels

**Figure 5:**
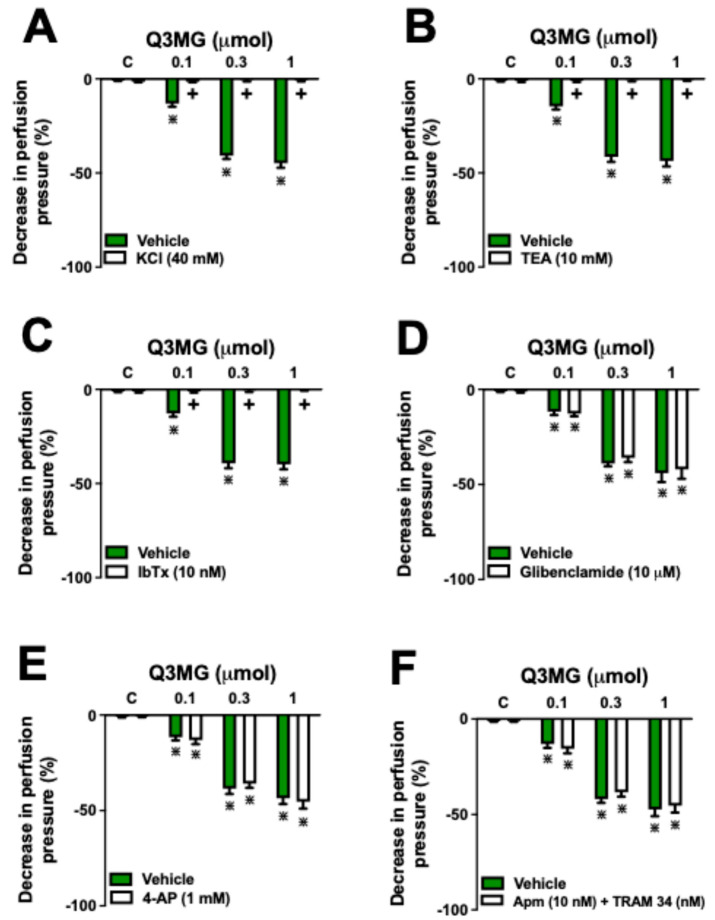
Role of K^+^ channels in the vasorelaxant effect of Q3MG. Effects of Q3MG on MVBs continuously perfused with 40 mM KCl (**A**), tetraethylammonium (TEA, (**B**)), iberiotoxin (IbTX, (**C**)), glibenclamide (**D**), 4-aminopyridine (4-AP, (**E**)), or apamin (Apm) plus TRAM-34 (**F**) are presented. The results show the mean ± S.E.M. of 6 preparations per group. The comparison between the different doses of Q3MG was conducted through one-way ANOVA followed by the Bonferroni test. The difference between the administration of inhibitors/antagonists and the perfusion with the vehicle only, at each dose, was performed using the Student’s *t*-test. **^+^** indicates *p* < 0.05 compared with the effects of Q3MG on the vehicle group. **^⋇^** indicates *p* < 0.05 compared with the respective control group. The vehicle represents the perfusion with only the physiological saline solution. All experiments were performed in the mesenteric vascular bed pre-contracted with PSS containing Phe 3 µM.

The perfusion of the MVBs with PSS containing 40 mM KCl and tetraethylammonium (a nonspecific K^+^ channel blocker) reduced the vasodilatory effect induced by Q3MG doses (Figure 5A,B) by 97 ± 2% and 98 ± 3%, respectively. Similarly, the previous iberiotoxin (IbTX) perfusion, a selective large-conductance, calcium-activated K^+^ channel (BK_Ca_) blocker, reduced the Q3MG vasodilatory response by 93 ± 2% (Figure 5C). On the other hand, previous infusion with glibenclamide (an ATP-sensitive K^+^ channel blocker), 4-aminopyridine (a voltage-gated K^+^ channel blocker), or apamin (a selective, small-conductance, calcium-activated K^+^ channel (SK_Ca_) blocker) plus TRAM-34 (a selective intermediate-conductance, calcium-activated K^+^ channel (IK_Ca_) blocker) did not affect the vasorelaxant effects of Q3MG (Figure 5D–F).

### 2.5. Effects of Q3MG on Intracellular Concentration of Cyclic Guanosine Monophosphate (cGMP)

**Figure 6:**
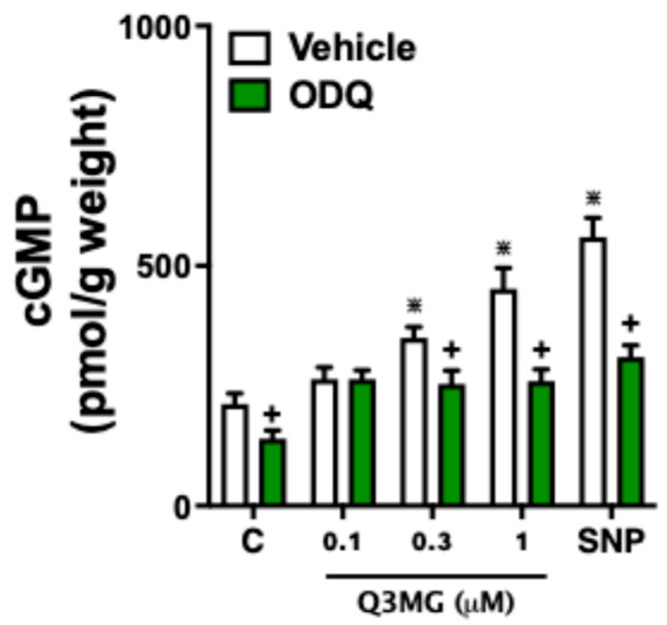
Effects of Q3MG on intracellular cGMP levels. Spontaneously hypertensive rat aortic rings incubated with Q3MG (0.1, 0.3, and 1 µM) or sodium nitroprusside (SNP) in the absence and presence of ODQ (100 µM) are shown. The results show the mean ± S.E.M. of 5 preparations per group. **^+^** indicates *p* < 0.05 compared with the effects of the respective group in the presence of ODQ. **^⋇^** indicates *p* < 0.05 compared to the control group in the absence of ODQ.

Incubation of Q3MG (0.3 and 1 µM) with the aortic rings of SHR rats increased the cGMP levels by approximately 42% and 83%, respectively, compared to basal levels, whereas its co-incubation with ODQ (100 μM) completely abolished this effect. The NO donor SNP increased the cGMP levels by approximately 96%, whereas co-incubation with ODQ completely eliminated the SNP-mediated increases in cGMP (Figure 6).

## 3. Discussion

Several studies have already shown that different flavanols, a class of flavonoids similar to Q3MG, can trigger the release of various endothelial mediators, including NO, prostacyclin (PGI2), and the endothelial-derived hyperpolarizing factor (EDHF), which induces arteriolar vasodilation and decreases arterial pressure [14,15,16]. Thus, we employed molecular docking as a computational tool to predict the binding affinities and interactions of Q3MG with vascular target proteins. Our results reveal that both classical and weaker non-covalent interactions work together to confer high affinity and selectivity in the Q3MG–1M9M and Q3MG–6ND0 complexes. In the eNOS (1M9M) active site, multiple conventional hydrogen bonds with residues ALA446, CYS99, SER102, and ASN466 lock the ligand into optimal conformation for catalytic guanylate cyclase activation. The thermodynamic contribution of hydrogen bonds to ligand binding—accounting for roughly 20–40% of binding energy—has been detailed by Desiraju et al. [17]. In addition, π–π stacking with TRP445 and π–alkyl contacts involving ALA446 and CYS99 are consistent with Meyer and Diederich’s [18] finding that aromatic interactions yield significant entropic gains by embedding ligand rings into the hydrophobic pocket.

In the 6ND0 binding mode, an array of hydrogen bonds with polar residues ARG578, ARG996, GLU569, and ASP482 establishes a robust polar interface between the ligand and the RCK domains of the channel. Repeated π–alkyl interactions around PRO1005 further stabilize hydrophobic regions within the lipid bilayer, facilitating channel opening. π–Sigma contacts that constrain intraligand ring vibrations—and thus improve binding kinetics—are in accordance with Tonggu and Wang [19]. Collectively, this combinatorial network of interactions provides the thermodynamic and conformational foundation for Q3MG’s mechanically induced BK_Ca_ channel opening [20].

The most striking finding of this study is the binding energy of −9.4 kcal/mol observed for Q3MG bound to 6ND0. Under the same docking protocol, β-sitosterol and L-citrulline demonstrated binding energies of −7.0 kcal/mol [21] and −5.4 kcal/mol [22], respectively, highlighting Q3MG’s markedly higher affinity. This comparison underscores the superior thermodynamic and kinetic stability of the Q3MG–6ND0 complex. Q3MG also exhibits exceptional efficiency metrics relative to its molecular size, with ligand efficiency (LE) values of 0.238–0.241, fit quality (FQ) of 0.829–0.838, and binding efficiency index (BEI) of 0.016–0.017. Estimated Ki values (0.128–0.151 µM) further support its high affinity and potential for effective modulation at low doses. These parameters suggest that Q3MG is not only a potent binder but also a promising candidate in terms of bioavailability and toxicological profile. Despite methodological differences among docking platforms, reference compounds such as benzoxazolinone–triazole derivatives (−4.09 kcal/mol) and indomethacin (−3.83 kcal/mol), reported with Glide by Haider et al. [23], further underscore Q3MG’s exceptional potential. In summary, Q3MG emerges as a highly promising dual-mode natural modulator, simultaneously activating eNOS and promoting BK_Ca_ channel opening with exceptional binding affinity and interaction efficiency.

Based on the mapping of possible molecular targets of Q3MG, we investigated its vascular effects in MVBs from SHR rats, where a possible alteration in the expression of eNOS and BK_Ca_ channels induced by hypertension would already be established [24,25]. The vascular endothelium regulates the tone of resistance vessels, directly influencing blood pressure control. This effect is regulated by various autacoids, among other factors, that control vascular homeostasis in response to chemical or physical stimuli, including shear stress and pulsatile stretching [26]. The main mediators derived from the vascular endothelium are NO, PGI2, and EDHF, which, by activating the NO/cGMP and PGI2/cAMP pathways, as well as the opening of K_Ca_ channels, play a crucial role in the control of the tone of pre-capillary arterioles [27,28]. Our data demonstrate that the vascular endothelium plays a direct role in the vasodilatory response of Q3MG, as the chemical removal of the endothelium completely blocked the vasodilatory response. Furthermore, we showed that NO has a central role in this effect, as prior use of L-NAME, a NO synthesis inhibitor, apart from blocking the activity of guanylate cyclase with methylene blue, completely inhibited the vasodilatory response of Q3MG. Considering that the vasodilatory effects of NO through the activation of guanylate cyclase involve the increase in cGMP levels [29], and that plants rich in flavonoids increase intracellular levels of cGMP [30], we chose to investigate whether the incubation of arterial tissue with Q3MG would be able to increase cGMP levels. The data showed that Q3MG was able to increase the intracellular levels of cGMP, confirming a direct relationship with the activation of the NO/cGMP pathway.

The downstream targets of the NO/cGMP pathway in resistance vessels are known to involve the opening of K^+^ channels, influencing the maintenance of arteriolar tone. Additionally, previous data indicate that flavonoids can induce the opening of K^+^ channels in vascular smooth muscle through the NO/cGMP pathway [31,32]. To investigate whether K^+^ channels play a central role in the vasodilatory effects induced by Q3MG, we chose two different approaches. First, we used the perfusion of a solution with a high concentration of KCl (40 mM), which suppresses the K^+^ current through smooth muscle fibers [33]. Then, we utilized tetraethylammonium, which non-specifically blocks K^+^ channels [34,35]. In both approaches, the effects of Q3MG on the MVBs were completely inhibited, demonstrating that the effects of this flavonoid are dependent on the opening of K^+^ channels in arteriolar smooth muscle.

Different K^+^ channels, including ATP-sensitive, voltage-gated, and K_Ca_ channels, participate in the vasodilatory response to chemical mediators or electrical and physical stretches [31,36]. As a previous perfusion of blockers of ATP-dependent (glibenclamide) or voltage-dependent K^+^ channels (4-aminopyridine) did not affect the vascular effect of Q3MG, we investigated the role of K_Ca_ channels, perfusing highly selective blockers of SK_Ca_, IK_Ca_, and BK_Ca_, i.e., apamin, TRAM-34, and IbTX, respectively. Only the prior perfusion of IbTX was able to abolish the vasodilatory response to Q3MG, suggesting that BK_Ca_ plays a crucial role in the reduction of peripheral resistance in MVBs of SHR rats.

Few studies have described the pharmacological effects of Q3MG on the cardiovascular system. Plants rich in Q3MG have shown antioxidant [11], antiatherogenic [12], and hypoglycemic [13] activities; however, the effects on peripheral vascular resistance remain unknown. Our study provides, for the first time, evidence that Q3MG has vasodilatory actions in resistance vessels, providing a fundamental understanding to support future research on in vivo models and eventually lead to controlled clinical trials.

Despite the relevance of our findings, we highlight two important limitations in our study. The first is that we do not know how Q3MG would behave when used in vessels from normotensive animals. This information would be useful for us to adequately understand the effect of hypertension on the vascular effects induced by Q3MG. Another limitation was not fully elucidating the molecular mechanisms involved in the release of NO, as well as determining whether the effects on BK_Ca_ are direct or indirectly induced by the activation of eNOS. Future research may clarify these points and support the conduction of studies that evaluate the efficacy and safety of Q3MG, including its bioavailability after oral administration.

## 4. Materials and Methods

### 4.1. In Silico Experiments

#### Molecular Docking

Molecular docking was employed to elucidate the potential vasodilatory effects of Q3MG at the molecular level. This computational approach enables the prediction of ligand interactions with biological target proteins based on binding energies and conformational compatibility, providing a rational basis for further experimental validation. Specifically, endothelial nitric oxide synthase (eNOS; PDB ID: 1M9M) and the large-conductance, calcium-activated potassium channel (BK_Ca_; PDB ID: 6ND0) were selected as target proteins due to their central roles in the regulation of vascular tone and endothelial function. These protein structures were retrieved from the RCSB Protein Data Bank (PDB) and processed using BIOVIA Discovery Studio Visualizer 2021.

Pre-docking preparation involved the removal of crystallographic water molecules and non-standard heteroatoms, followed by the addition of polar hydrogen atoms to improve docking accuracy. Binding sites were defined based on the coordinates of the co-crystallized ligands within each protein structure using the “Define and Edit Binding Site—From Current Selection” tool in Discovery Studio. The refined protein structures were saved in .pdb format and subsequently converted to .pdbqt format using AutoDock Tools (version 1.5.7).

Native ligands were extracted from their respective complexes and saved in .pdb format before conversion to .pdbqt format. Test ligands were subjected to energy minimization to ensure optimal geometry and were similarly converted to .pdbqt format. Molecular docking simulations were then conducted, and multiple binding poses were evaluated. The conformation with the lowest binding energy (i.e., most favorable docking score) was selected for further analysis. Corresponding output and log files were generated to support post-docking evaluation.

Protein–ligand interaction profiling was performed using BIOVIA Discovery Studio Visualizer 2021, allowing detailed analysis of hydrogen bonds, hydrophobic interactions, and other non-covalent contacts within the binding pocket, in accordance with the methodology described by Trott and Olson [36].

### 4.2. Pharmacological Assays

#### 4.2.1. Drugs and Reagents

The following drugs, salts, and solutions were used: xylazine and ketamine hydro-chloride (Syntec, São Paulo, SP, Brazil); heparin (Hipolabor Pharmaceutical, Belo Horizonte, MG, Brazil); sodium deoxycholate, Q3MG, acetylcholine chloride, phenylephrine, indomethacin, glibenclamide, Nω-Nitro-L-arginine methyl ester (L-NAME), tetraethylammonium bromide, iberiotoxin, TRAM-34, apamin, NaCl, KCl, NaHCO_3_, MgSO_4_, CaCl_2_, KH_2_PO_4_, dextrose, and ethylenediaminetetraacetic acid (EDTA) (Sigma-Aldrich, Saint Louis, MO, USA).

#### 4.2.2. Animals

Fourteen-week-old male spontaneously hypertensive rats (SHR; 300–320 g) were provided by the central animal facility at the Federal University of Grande Dourados (UFGD, Brazil). The animals were given food and water freely under controlled conditions, including a temperature of 22 ± 2 °C, humidity of 50 ± 10%, and a 12 h light/12 h dark cycle (lights on at 07:00 AM). All experimental procedures carried out in this study were conducted in accordance with the Guidelines for the Care and Use of Laboratory Animals as adopted and promulgated by the U.S. National Institutes of Health. Furthermore, this study was approved by the Institutional Ethics Committee of UFGD (protocol code 23011, 24 July 2023).

#### 4.2.3. Isolation and Preparation of the Mesenteric Vascular Beds (MVBs)

The animals were anesthetized with ketamine and xylazine (100 and 20 mg/kg, respectively) via the intraperitoneal route. Then, the MVBs were prepared as previously described [37]. The MVBs were retired and placed in a water-jacketed organ bath. The preparations were maintained at 37 °C and perfused at 4 mL/min with physiological saline solution (PSS; composition in mM: NaCl 119, KCl 4.7, CaCl_2_ 2.4, MgSO_4_ 1.2, NaHCO_3_ 25.0, KH_2_PO_4_ 1.2, dextrose 11.1, and EDTA 0.03; pH 7.4) gassed with 95% O_2_/5% CO_2_. All preparations were equilibrated for 30 to 45 min, and their viability was checked by an injection of KCl (120 mmol). Changes in the perfusion pressure (mm Hg) were recorded by a pressure transducer connected to an acquisition system (PowerLab^®^) and its application program (Chart, v 7.1; both from ADI Instruments, Castle Hill, Australia).

#### 4.2.4. Investigation of the Vasodilatory Effects of Q3MG and the Molecular Mechanisms Involved

Initially, MVBs with functional endothelium were continuously perfused with PSS plus phenylephrine (3 µM). After stabilizing the contraction response, to verify the viability and functional integrity of the preparations, we administered a bolus injection of 1 nmol of ACh, and the vasodilatory response was recorded. Additionally, we administered a dose of the PSS in all the pre-contracted preparations to demonstrate the absence of any vasodilatory response induced by the vehicle used in the experiments. Subsequently, preparations received injections containing 0.01, 0.03, 0.1, 0.3, and 1 µmol of Q3MG, and the perfusion pressure was recorded. Each subsequent administration was performed with an interval of 3 min between doses.

To investigate the molecular mechanisms involved in the vasodilatory effects induced by Q3MG, we used the methodology proposed by Klider et al. [38]. First, to assess the effects in the physical absence of the endothelium, different preparations were perfused with PSS plus sodium deoxycholate (1.8 mg/mL) for 30 s. The reliability of sodium deoxycholate to promote endothelium removal was verified by the lack of reduction in the perfusion pressure after a bolus injection of 1 nmol ACh. Subsequently, a serial administration with Q3MG (0.1, 0.3, and 1 µmol) was performed.

Using preparations with intact endothelium, different MVBs were perfused with PSS plus 3 µM Phe, given alone or combined as follows: L-NAME (100 μM; a non-selective nitric oxide synthase inhibitor), indomethacin (1 µM; a non-selective inhibitor of cyclooxygenases), MB (100 μM; an inhibitor of guanylate cyclase (GC)), KCl (40 mM), tetraethylammonium (10 mM; a nonspecific K^+^ channel blocker), glibenclamide (10 µM; an ATP-sensitive K^+^ channel blocker), 4-aminopyridine (100 μM; a voltage-gated K^+^ channel blocker), IbTX (10 nM; a selective BK_Ca_ blocker), TRAM-34 (a selective IK_Ca_ blocker), and apamin (10 nM; a selective SK_Ca_ blocker). After 15 min of stabilization of the preparations, Q3MG (0.1, 0.3, and 1 µmol) was again injected into the perfusion system. The ability of the flavonoid to reduce the perfusion pressure was compared with the results obtained in preparations perfused with vehicle only.

#### 4.2.5. Effects of Q3MG on the Intracellular Concentration of Cyclic Guanosine Monophosphate (cGMP)

To evaluate the effects of Q3MG on the intracellular concentration of cGMP, we utilized the methods described by Estancial et al. [39]. First, aortic rings of male SHR rats (2–3 mm; n = 5) were removed and mounted in an organ bath with Krebs–Henseleit solution (composition in mM: 117 NaCl, 4.7 KCl, 2.5 CaCl_2_, 1.2 MgSO_4_, 1.2 KH_2_PO_4_, 25 NaHCO_3_, and 11 glucose) at 37 °C and gassed with 95% O_2_/5% CO_2_. A resting period of 1 h, under a tension of 2 g, was allowed before experiments. Then, aortic rings were incubated for 15 min with SNP (10 µM) or Q3MG at different concentrations (0.1, 0.3, and 1 µM) in the absence and presence of GC inhibitor ODQ (100 µM, 30 min). Then, tissues were removed, frozen, homogenized in trichloroacetic acid (5% wt/vol), centrifuged (10 min at 4 °C at 1500× *g*), and the supernatant was collected. Methods for antibody incubation and the measurement of intracellular cGMP were performed as described in commercially available kits (Cayman Chemical Cyclic GMP EIA kit, Ann Arbor, MI, USA). All experiments were performed in triplicate.

### 4.3. Statistical Analysis

The data are expressed as the mean ± standard error of a mean of six preparations per group. Statistical analyses were performed using one-way analysis of variance (ANOVA) followed by Bonferroni’s test or Student’s *t*-test, when applicable. A *p*-value of less than 0.05 was considered statistically significant. Graphs were drawn and statistical analyses were performed using GraphPad Prism 10 for macOS (GraphPad Software, Boston, MA, USA).

## 5. Conclusions

This study demonstrated that the flavonoid Q3MG can induce endothelium-dependent vasodilation in the resistance arteries of hypertensive rats. The data obtained from molecular docking and through studies in isolated and perfused MVBs allowed us to suggest that the vascular effects of Q3MG involve the activation of the NO/cGMP pathway followed by the opening of BK_Ca_. Further research is needed to evaluate the clinical implications of these findings and to lead to a better understanding of the potential uses of Q3MG in the prevention and treatment of cardiovascular diseases.

## Figures and Tables

**Table 1:** Docking scores and report of predicted interactions of docked conformations of Q3MG against the binding sites of both 1M9M and 6ND0 proteins.

Protein	H-Bond	Pi–Pi Stacking	Alkyl Interactions
1M9M	Conventional H-bonds: ALA446:HN–O5, CYS99:HG–O12, SER102:HG–O3, H1–ALA443:O, H2–ALA443:O, H3–O7, H4–GLY101:O, H4–O7, H8–SER102:O, H17–ASN466:O C–H bonds: GLY101:HA2–O7, SER102:HB1–O4, H13–O7, H21–ASN466:OD1 Pi–Donor H-bond: TRP445:HN–Ligand	TRP445 (×2 Pi–Pi stacked)	ALA446, CYS99 (Pi–Alkyl)
6ND0	Conventional H-bonds: ARG578:HH12–O15, ARG996:HH12–O7/O8, ARG996:HH21–O8, H1–GLU569:OE2, H4–ASP482:OD1, H4–O7, H5–GLU1008:OE1, H8/H9–SER577:O, H17–ILE579:O C–H bonds: GLU576:HA–O11, ARG578:HA–O14, H15–GLU569:OE2, H21–O14	Pi–Sigma (Ligand internal)	PRO1005 (×3) (Pi–Alkyl)

## Data Availability

The original contributions presented in this study are included in the article. Further inquiries can be directed to the corresponding author(s).

## Abbreviations

CVDs	Cardiovascular Diseases
Q3MG	Quercetin 3-O-MalonylGlucoside
eNOS	Endothelial Nitric Oxide Synthase
BK_Ca_	Large-Conductance Calcium-Activated Potassium Channel
MVBs	Mesenteric Vascular Beds
cGMP	Cyclic Guanosine Monophosphate
ACh	Acetylcholine
L-NAME	Nω-Nitro-L-Arginine Methyl Ester (NO Synthase Inhibitor)
PGI_2_	Prostacyclin
EDHF	Endothelium-Derived Hyperpolarizing Factor
TEA	Tetraethylammonium (Non-Selective K^+^ Channel Blocker)
IbTX	Iberiotoxin (Selective BK_Ca_ Blocker)
PSS	Physiological Saline Solution
SHR	Spontaneously Hypertensive Rats
FQ	Fit Quality
LE	Ligand Efficiency
BEI	Binding Efficiency Index
Ki	Inhibition Constant
ANOVA	Analysis of Variance
ATP	Adenosine Triphosphate
SK_Ca_	Small-Conductance Calcium-Activated Potassium Channel
IK_Ca_	Intermediate-Conductance Calcium-Activated Potassium Channel
PDB	Protein Data Bank
MB	Methylene Blue
TRAM-34	Selective IK_Ca_ Channel Blocker
Apm	Apamin (Selective SK_Ca_ Channel Blocker)
